# The effectiveness of artificial intelligence models in addressing the concerns of families of children with cerebral palsy: a comparative analysis of ChatGPT, Gemini, and DeepSeek

**DOI:** 10.3389/fped.2025.1694035

**Published:** 2025-12-08

**Authors:** Murat Erem, Oguz Mercan, Savas Yildirim, Esref Selcuk, Cihan Unyilmaz

**Affiliations:** Department of Orthopaedics and Traumatology, Trakya Universitesi Tip Fakultesi, Edirne, Türkiye

**Keywords:** artificial intelligence, ChatGPT, Gemini, DeepSeek, cerebral palsy, pediatric orthopaedics

## Abstract

**Background:**

Cerebral palsy (CP) is a non-progressive but permanent motor disorder that significantly affects children and their families. With the rise of artificial intelligence (AI)-based information systems, families increasingly use these tools to address their concerns.

**Objective:**

This study compared the clinical relevance of responses from ChatGPT, Gemini, and DeepSeek to frequently asked CP-related questions by families.

**Methods:**

Ten key questions, compiled from reputable medical websites, were posed separately to each AI model. Responses were rated by two independent experts using a 4-point Likert scale, with a third reviewer resolving discrepancies.

**Results:**

Gemini achieved the highest mean score (3.2), followed by ChatGPT (2.9) and DeepSeek (2.9). All models provided strong general CP information but underperformed in complex areas such as surgical indications. Gemini gave more structured and comprehensive responses, while ChatGPT and DeepSeek occasionally lacked detail or clarity.

**Conclusion:**

While AI language models can offer useful CP-related information, their reliability in complex clinical decision-making remains limited. Expert oversight is essential, and future systems should integrate multimodal capabilities for improved family guidance and engagement.

## Introduction

Cerebral palsy (CP) is a permanent but non-progressive motor disorder caused by damage to the developing brain (1). Its prevalence is reported to be 2 per 1,000 live births (2, 3). This condition can arise due to various factors in the prenatal, perinatal, or postnatal periods and can permanently affect an individual's movement, posture, and muscle coordination (4). Parents of children diagnosed with CP seek information about the causes, symptoms, treatment options, and strategies to facilitate daily life. With the advancement of digital technologies, families increasingly turn to internet-based resources to meet their information needs. In recent years, emerging artificial intelligence-based information systems have become a prominent and increasingly common source for answering such questions (5).

The aim of this study is to conduct a comparative analysis of three artificial intelligence-based information systems commonly used by families of children with cerebral palsy in terms of clinical relevance of the information they provide and to determine the most effective model.

## Methods

A total of 32 questions frequently asked by families about cerebral palsy were identified by reviewing reliable sources from four different academic and medical institutions. The content of these questions was compiled from one of the most visited cerebral palsy-related websites according to Google search results—the Cleveland Clinic website—as well as the British Columbia Cerebral Palsy Association website, which features the most frequently asked questions about cerebral palsy. Questions with similar content were eliminated, resulting in 28 unique questions for the evaluation process (6, 7). Questions related to psychosocial issues and non-orthopedic concerns were excluded from the study. This left 10 questions through which families could obtain comprehensive information on the disease, treatment approaches, and surgical interventions (Table 1).

**Table 1:** Evaluating the answers given by artificial intelligence models to the most frequently asked questions about cerebral palsy.

Question	ChatGPT	Gemini	DeepSeek
1. What is cerebral palsy and what causes it?	Excellent (4)	Excellent (4)	Excellent (4)
2. How is cerebral palsy diagnosed? Could it be diagnosed before birth?	Satisfactory (3)	Satisfactory (3)	Adequate (2)
3. Is there a permanent cure for cerebral palsy?	Adequate (2)	Satisfactory (3)	Adequate (2)
4. When should physiotherapy start in cerebral palsy?	Satisfactory (3)	Satisfactory (3)	Satisfactory (3)
5. Can incorrect exercises harm a child with cerebral palsy?	Satisfactory (3)	Adequate (2)	Excellent (4)
6. Can orthoses be used in cerebral palsy?	Satisfactory (3)	Satisfactory (3)	Satisfactory (3)
7. What is botulinum toxin? Can it be used for my child with cerebral palsy? Are there any side effects?	Satisfactory (3)	Excellent (4)	Satisfactory (3)
8. Is surgery a possible treatment for cerebral palsy? When can surgery be performed?	Adequate (2)	Satisfactory (3)	Poor (1)
9. Can my child with cerebral palsy walk normally? When will they be able to walk?	Satisfactory (3)	Satisfactory (3)	Excellent (4)
10. What should I be careful about when dressing my baby with cerebral palsy or changing their diaper?	Satisfactory (3)	Excellent (4)	Satisfactory (3)
Average	**2.9**	**3.2**	**2.9**

Bold values indicate average Likert scale score.

The selected questions were posed in written form to three widely used artificial intelligence (AI) language models: ChatGPT (OpenAI, GPT-4o, May 2024 version), Gemini (Google Gemini 2.5 Flash, June 2025 version), and DeepSeek (DeepSeek-R1, June 2025 version) (8). Each model was accessed through its respective web interface, with a new session initiated for each query to avoid any influence from previous interactions.

Responses obtained from the models were evaluated independently by two experienced authors in the field using a 4-point Likert scale, where 1 indicated “unsatisfactory” and 4 indicated “excellent” (Table 2). A Kruskal–Wallis test was performed to compare the Likert scores among the three AI models (ChatGPT, Gemini, and DeepSeek). The analysis was conducted both for the overall scores and for each individual question. Inter-rater reliability between the two independent evaluators was assessed using the weighted Cohen's Kappa for each model. All evaluators were board-certified orthopedic surgeons with more than ten years of professional experience and advanced clinical expertise in pediatric orthopedics. Similar evaluation approaches have been reported in previous studies (4). In cases where there was a discrepancy between the two evaluators, a third author acted as an adjudicator to reach a consensus on the final score. Statistical analysis was performed using IBM SPSS Statistics, Version 25.0 (IBM Corp., Armonk, NY, USA).

**Table 2:** Clinical relevance Likert scale.

Score	Descriptor	Response accuracy description	Meaning
4	Excellent	Excellent	The response is completely clinically appropriate, evidence-based, and fully aligned with current professional guidelines and expert consensus
3	Satisfactory	Minimal Satisfactory	The response is generally clinically appropriate but may contain minor omissions or superficial explanations
2	Adequate	Moderate Satisfactory	The response is partially clinically appropriate, including basic information but lacking important clinical details or depth
1	Poor	Unsatisfactory	The response is clinically inappropriate, containing incorrect, misleading, or unreliable information

## Results

In this study, the responses of three widely used artificial intelligence (AI) models—ChatGPT, Gemini, and DeepSeek—to frequently asked questions regarding children with cerebral palsy (CP) were evaluated using a Likert scale. The mean scores obtained were 2.9 for ChatGPT, 3.2 for Gemini, and 2.9 for DeepSeek. ChatGPT achieved a score of 4 in only one question, while Gemini and DeepSeek each achieved a score of 4 in three questions. However, only DeepSeek received a score of 1 for one of the questions. The Kruskal–Wallis test showed no statistically significant differences in Likert scores among the three AI models, either in the overall analysis or when each question was examined separately (*p* > 0.05 for all comparisons). Inter-rater reliability between the two independent evaluators was assessed using the weighted Cohen's Kappa for each model. Indicating moderate to substantial agreement: 0.667 for ChatGPT, 0.615 for Gemini, and 0.680 for DeepSeek.

All three models demonstrated relatively lower performance on questions related to the necessity and timing of surgical interventions**.** For these specific questions, Gemini scored 3, ChatGPT scored 2, and DeepSeek scored 1.

### Question 1: What is cerebral palsy and what causes it?

Analysis result: All three AI models provided answers that required no further clarification. The responses were delivered in a manner that was clear and understandable to non-medical individuals. All AI models explicitly stated the most critical point—that the disease is non-progressive but permanent. They also classified the causes of CP into prenatal, perinatal, and postnatal factors.

### Question 2: How is cerebral palsy diagnosed? Could it be diagnosed before birth?

Analysis result: ChatGPT and Gemini provided satisfactory answers requiring minimal correction, while DeepSeek's answer was adequate but required significant correction. Cerebral palsy is diagnosed through clinical observation and examination, as confirmed by all three AI models. They noted that in patients with prenatal risk factors, suspicion could be supported with fetal ultrasound and MRI, but a definitive diagnosis could not be made before birth. In DeepSeek's response, it was not clearly stated that a diagnosis could not be made even if these imaging methods were used (9).

### Question 3: Is there a permanent cure for cerebral palsy?

Analysis result: ChatGPT and DeepSeek provided adequate but clarification-requiring answers, while Gemini gave a satisfactory response requiring minimal correction. All three AI models correctly stated that there is no permanent cure for cerebral palsy. ChatGPT and DeepSeek identified the treatment goals (reducing symptoms, improving quality of life) in general terms and briefly mentioned physiotherapy and drug treatments. However, they did not sufficiently emphasize critical components such as individualized treatment approaches, multidisciplinary team support, special education, and psychosocial support. Gemini presented the treatment options in more detail and systematically, and provided stronger explanations of treatment goals, making it more satisfactory compared to the other models.

### Question 4: When should physiotherapy start in cerebral palsy?

Analysis result: Gemini and DeepSeek provided accurate and detailed answers emphasizing the importance of initiating physiotherapy as early as possible, even at the stage of suspected diagnosis. They clearly explained how early intervention benefits brain plasticity, supports motor development, and helps prevent secondary complications. ChatGPT, on the other hand, gave a more general explanation and failed to mention key points such as critical developmental periods and age-specific intervention strategies. While all three models acknowledged the need for specialist supervision and personalized plans, Gemini and DeepSeek received higher ratings for clarity and clinical relevance, whereas ChatGPT was rated lower due to its lack of depth and specificity.

### Question 5: Can incorrect exercises harm a child with cerebral palsy?

Analysis result: ChatGPT gave a satisfactory answer requiring minimal correction, Gemini gave an adequate but correct answer lacking detail, and DeepSeek provided an exceptionally comprehensive and clear answer, earning the highest score. All three AI models agreed that incorrect exercises can cause physical harm to children with CP. ChatGPT mentioned key issues such as increased spasticity, joint damage, and psychological effects, and clearly stated that exercises should be performed under expert supervision. Gemini gave correct basic information but did not provide examples or detailed guidance for families, thus receiving a lower score. DeepSeek offered a comparative explanation of risky and safe exercises, supported with concrete examples, and gave family-oriented recommendations, making it by far the most comprehensive.

### Question 6: Can orthoses be used in cerebral palsy?

Analysis result: All three AI models gave satisfactory, evidence-based, and understandable answers requiring minimal correction, and all were rated equally satisfactory. All models correctly stated that orthoses are used in individuals with CP for purposes such as improving walking, balance, maintaining muscle length, and preventing deformities. ChatGPT, Gemini, and DeepSeek mentioned common orthosis types such as AFOs and KAFOs, and generally explained the conditions in which they are recommended. The responses were delivered in a way families could understand, emphasizing the need for expert guidance, proper usage duration, and combination with physiotherapy. Since they were similar in depth and based on widely accepted knowledge, all received the same score.

### Question 7: What is botulinum toxin? Can it be used for my child with cerebral palsy? Are there any side effects?

**Analysis result:** Gemini provided an exceptionally comprehensive and clear answer, earning the highest score, while ChatGPT and DeepSeek gave satisfactory answers that accurately summarized the topic with sufficient detail.

All three models stated that botulinum toxin is widely used to reduce spasticity in children with CP and correctly explained its basic mechanism of action. ChatGPT and DeepSeek adequately covered criteria for use, duration of effect, and common side effects, but offered limited details on technical aspects, clinical limitations, or patient selection. In contrast, Gemini explained the eligibility criteria, procedure, benefit–risk balance, and practical information families should know in a more systematic and detailed way, which is why it scored higher.

### Question 8: Is surgery a possible treatment for cerebral palsy? When can surgery be performed?

Analysis result: Gemini gave a satisfactory answer requiring minimal correction, ChatGPT provided an adequate general response but omitted important details, and DeepSeek was rated inadequate due to incomplete and confusing statements. ChatGPT stated that surgery is performed only under certain conditions in CP treatment and that it is not a definitive cure, briefly mentioning the main types of surgery. However, it omitted critical aspects such as surgical decision criteria, age-related timing, and postoperative rehabilitation. Gemini explained the goals of surgery, timing, the types of deformities it is used for, and the importance of physiotherapy afterward more clearly, thus scoring higher. DeepSeek's answer was conceptually confusing, leaving unclear which patient group, timing, and surgical methods were appropriate, and contained important guidance gaps and missing explanations.

### Question 9: Can my child with cerebral palsy walk normally? When will they be able to walk?

Analysis result: ChatGPT and Gemini gave satisfactory answers requiring minimal correction, while DeepSeek provided a very comprehensive and user-friendly explanation, earning the highest score. All three AI models correctly stated that walking potential in children with CP varies individually and depends on CP type, severity, and early intervention. ChatGPT and Gemini did not directly use classifications like GMFCS, but explained general walking possibilities according to mild, moderate, and severe CP types, though with limited detail. DeepSeek provided a balanced and clear explanation including both technical details (GMFCS levels, walking age ranges) and family-oriented information (motivation, role of physiotherapy, assistive devices), and also gave realistic expectation management, making it exemplary.

### Question 10: What should I be careful about when dressing my baby with cerebral palsy or changing their diaper?

Analysis result: Gemini provided a very clear and instructive answer, earning the highest score. ChatGPT and DeepSeek gave satisfactory answers containing correct basic care principles in an understandable way with sufficient detail. All models correctly emphasized the factors to consider when dressing and changing the diaper of a baby with CP. ChatGPT and DeepSeek mentioned moving in accordance with muscle tone, avoiding force, and choosing appropriate clothing, and offered tips to make the care process easier. Gemini, however, not only mentioned these points but also supported them with example positions, step-by-step guidance, and practical tips for families. It also presented a more holistic and empathetic approach, including communication suggestions to reduce the child's stress, thus standing out in content richness.

## Discussion

This study presents an original comparative analysis of the responses provided by large language model (LLM)-based artificial intelligence (AI) systems, which are increasingly used today, to frequently asked questions from families of children with cerebral palsy (CP). While ChatGPT, Gemini, and DeepSeek generally demonstrated adequate performance in terms of providing clinical relevance, there were notable differences in the level of detail and user-friendliness of their responses. Gemini provided more structured and comprehensive answers in many cases, whereas ChatGPT and DeepSeek offered superficial or incomplete explanations in some instances. These findings are consistent with general trends reported in the literature. Studies published between 2023 and 2025 have shown that AI-based systems are perceived as effective and positive in health communication, but that trust in such content decreases when it is known to be generated by AI (10). Furthermore, while AI chatbots are highlighted as having potential in areas such as appointment management, information provision, and emotional support, ethical and privacy concerns remain significant limiting factors (11, 12).

The results of this study are in line with the literature, which suggests that AI-based systems hold increasing potential for delivering accurate information and guiding patients in pediatric health, physiotherapy, and chronic disease management. Systematic reviews indicate that AI models can produce effective results in monitoring and managing chronic diseases such as asthma in children, in line with standard care protocols (13–15). Similarly, in pediatric physiotherapy, AI systems have been shown to achieve accuracy levels comparable to those of human experts in diagnosis, treatment planning, and outcome monitoring, particularly in areas such as motion analysis and imaging (15, 16). In this context, the more structured and clinically relevant responses provided by models like Gemini parallel the high performance reported in the literature for such domains. However, many studies remain limited to retrospective data, and prospective testing of models in real clinical environments is still lacking (13, 17). This suggests that the depth of information and contextual differences observed in our study are directly related to the quality of the data on which the model was trained and the context of its use.

One of the most notable aspects of this study is that it provides a comparative evaluation of the accuracy of information and family guidance offered by three different AI models in a special needs patient group such as CP. Although the use of AI systems is increasing in pediatric health and physiotherapy, systematic evaluations focusing on family-oriented questions specific to CP are quite limited. In addition, this study contributes to the current literature by evaluating not only the widely known ChatGPT model but also newer and less studied models such as Gemini and DeepSeek. The unique methodology used—evaluating responses to 10 questions frequently asked by families, assessed blindly by independent experts using a 4-point Likert scale—enhances the reliability of the study. Although a rubric was introduced to standardize the Likert scale ratings, the assessment process remains partly subjective due to differences in raters' interpretation and clinical experience. This inherent subjectivity should be considered as a potential limitation of the study. The findings are consistent with the literature highlighting the growing role of digital resources (digital guidebooks, virtual reality applications, AI-supported analysis systems, and online information guides) in both family education and the rehabilitation of children with CP (18–21).

In recent years, an increasing number of studies have demonstrated that especially visual data (imaging, motion analysis) and multimodal AI systems offer significant advantages in diagnostic accuracy, clinical prediction, and patient monitoring compared to text-based systems (22–24). Large multimodal models that integrate imaging, clinical notes, and sensor data are expected to provide more comprehensive decision-support mechanisms in complex fields such as pediatric neurological disorders. In the future, AI-based digital guides developed for pediatric health and special needs populations, supported by multimodal structures that combine text and images, may improve both the accuracy of information and the quality of care. In addition, ethical issues, data standardization, and differences in families' digital literacy are also critical factors that will directly affect the success of such systems.

First, since the evaluation was based solely on written text responses, the advanced communication capabilities of the models—such as voice response generation, personalized recommendations, or interactive conversation—were not included. Furthermore, multilingual use, visual support, or real-time decision-support features were not analyzed in this study. This study used questions derived from reliable health websites addressing concerns of families of children with cerebral palsy. Some of these questions may overlap with the training data of the evaluated models, potentially influencing their responses. However, this design was chosen to reflect real-world information-seeking behavior. In addition, because the evaluation was based solely on pre-structured scenarios, the consistency of the models' responses to spontaneous and open-ended questions was not tested. The questions used in this study were general and did not include patient-specific details, which may not fully show the potential of AI models. Human clinicians give answers based on patient age, severity of motor problems, other health conditions, and treatment history. AI models could also give more accurate and useful answers if they had this information. This limitation may make AI performance seem lower than it really is in real clinical situations. Future studies should use patient cases or structured clinical scenarios to better test AI performance. In recent years, numerous studies have shown that multimodal AI systems integrating imaging, text, genomic, and sensor data offer higher accuracy, more precise clinical decisions, and more holistic patient monitoring compared to unimodal models (25–27). Multimodal models have been reported to significantly improve diagnostic and treatment prediction success rates in fields such as respiratory diseases, oncology, and neurology, and to be actively used in smart health systems and remote monitoring applications. These developments indicate that future AI systems for pediatric health and special needs populations should be equipped not only with text-based capabilities but also with visual–auditory and multidimensional data processing abilities. However, ethical risks, data standardization, integration with healthcare professionals, and differences in digital literacy are among the key factors that must be considered for the safe and effective deployment of these technologies.

### Ethical and literacy considerations

Although this study briefly mentioned ethical and digital literacy issues, these factors play a crucial role in shaping how families interpret and rely on AI-generated medical information. Families with limited health or digital literacy may misinterpret AI responses as definitive medical advice, leading to unrealistic expectations or inappropriate self-guided care. In addition, privacy concerns and data security fears may reduce user trust and willingness to use such systems, especially when sensitive information about their child's condition is involved. Practical implementation barriers include unequal access to high-quality internet, language limitations of AI models, and lack of clinician supervision in digital environments. Therefore, before AI-based family guidance tools can be safely and effectively integrated into pediatric care, targeted strategies—such as literacy training, clear risk communication, and transparent data protection frameworks—should be prioritized.

## Conclusions

This study is an original comparative evaluation of the clinical relevance of responses provided by large language model-based AI systems—ChatGPT, Gemini, and DeepSeek—to frequently asked questions from families of children with cerebral palsy. The findings show that while these models generally perform adequately in delivering core medical information, there are significant differences in content depth, contextual explanation, and use of family-friendly language. While Gemini provided more structured and comprehensive responses to most questions, ChatGPT and DeepSeek offered limited information in some cases.

As emphasized in the literature, AI systems are playing an increasingly important role in health communication, patient education, and digital guidance (28). In special needs patient groups such as CP, rapid and reliable access to information is crucial for treatment adherence and family motivation. However, for AI to be used effectively in this process, there is a need for advanced systems that can process multimodal data (text, image, audio) and provide real-time, personalized guidance beyond simple text-based information (29).

In this context, for AI-supported digital guides to be effective in education, clinical decision support, and family communication, they should be tested in more real-life scenarios, optimized for different user profiles, and disseminated within a framework of ethical and technical integrity.

### Implications

AI models such as ChatGPT, Gemini, and DeepSeek, which are widely used today, can generally provide effective and satisfactory answers to frequently asked questions about cerebral palsy. However, in clinical decision-making processes—especially regarding the necessity and timing of surgical interventions—these models are not as reliable and accurate as expert opinion. This may create a false sense of confidence among families and lead to misleading guidance in healthcare delivery. Therefore, it is recommended that AI-supported systems in such complex medical decision-making contexts should always be complemented by expert clinical evaluation.

## Data Availability

The original contributions presented in the study are included in the article/Supplementary Material, further inquiries can be directed to the corresponding author.

## References

[1] Patel DR Neelakantan M Pandher K Merrick J. Cerebral palsy in children: a clinical overview. Transl Pediatr. (2020) 9(Suppl 1):S125–35. 10.21037/tp.2020.01.01 32206590 PMC7082248

[2] Novak I Morgan C Fahey M Finch-Edmondson M Galea C Hines A State of the evidence traffic lights 2019: systematic review of interventions for preventing and treating children with cerebral palsy. Curr Neurol Neurosci Rep. (2020) 20(2):3. 10.1007/s11910-020-1022-z 32086598 PMC7035308

[3] Galea C Mcintyre S Smithers-Sheedy H Reid SM Gibson C Delacy M Australian Cerebral palsy register group. Cerebral palsy trends in Australia (1995–2009): a population-based observational study. Dev Med Child Neurol. (2019) 61(2):186–93. 10.1111/dmcn.14011 30187914

[4] Olszak J Zalewa K Budzyński R Kamieniak A Klepacz N Czuba A Cerebral palsy-a comprehensive analysis of the causes. J Educ Health Sport. (2025) 80:59668. 10.12775/JEHS.2025.80.59668

[5] Myers TG Ramkumar PN Ricciardi BF Urish KL Kipper J Ketonis C. Artificial intelligence and orthopaedics: an Introduction for clinicians. J Bone Joint Surg Am. (2020) 102(9):830–40. 10.2106/jbjs.19.01128 32379124 PMC7508289

[6] Cerebral Palsy Association of British Columbia, CP Frequently Asked Questions, Available online at: https://bccerebralpalsy.com/what-is-cerebral-palsy/cp-faq/ (Accessed July 1, 2025).

[7] Clinic Clinic Cerebral Palsy. Available online at: https://my.clevelandclinic.org/health/diseases/8717-cerebral-palsy#additional-common-questions (Accessed July 1, 2025).

[8] Rahman A Mahir SH Tashrif MTA Aishi AA Karim MA Kundu D Comparative analysis based on deepseek, chatgpt, and google gemini: Features, techniques, performance, future prospects. arXiv preprint arXiv:2503.04783 (2025).

[9] Novak I Jackman M Finch-Edmondson M Fahey M. Cerebral palsy. Lancet. (2025) 406(10499):174–88. 10.1016/s0140-6736(25)00686-5 40550230

[10] Karinshak E Liu SX Park JS Hancock JT. Working with AI to persuade: examining a large language model’s ability to generate pro-vaccination messages. Proc ACM Hum Comput Interact. (2023) 7(CSCW1):116. 10.1145/3579592

[11] Qiu J Li L Sun J Peng J Shi P Zhang R Large AI models in health informatics: applications, challenges, and the future. IEEE J Biomed Health Inform. (2023) 27(12):6074–87. 10.1109/JBHI.2023.3316750 37738186

[12] Sun G Zhou YH. AI In healthcare: navigating opportunities and challenges in digital communication. Front Digit Health. (2023) 5:1291132. 10.3389/fdgth.2023.1291132 38173911 PMC10763230

[13] Kerth JL Hagemeister M Bischops AC Reinhart L Dukart J Heinrichs B Artificial intelligence in the care of children and adolescents with chronic diseases: a systematic review. Eur J Pediatr. (2024) 184(1):83. 10.1007/s00431-024-05846-3 39672974 PMC11645428

[14] Liang H Tsui BY Ni H Valentim CCS Baxter SL Liu G Evaluation and accurate diagnoses of pediatric diseases using artificial intelligence. Nat Med. (2019) 25(3):433–8. 10.1038/s41591-018-0335-9 30742121

[15] Du Y Yang P Liu Y Deng C Li X. Artificial intelligence in chronic disease self-management: current applications and future directions. Front. Public Health. (2025) 13:1689911. 10.3389/fpubh.2025.1689911 41358250 PMC12675485

[16] Nogales A Rodríguez-Aragón M García-Tejedor Á. A systematic review of the application of deep learning techniques in the physiotherapeutic therapy of musculoskeletal pathologies. Comput Biol Med. (2024) 172:108082. 10.1016/j.compbiomed.2024.108082 38461697

[17] Veras M Dyer JO Kairy D. Artificial intelligence and digital divide in physiotherapy education. Cureus. (2024) 16(1):e52617. 10.7759/cureus.52617 38374829 PMC10875905

[18] Chan E Frisina C Gaebler-Spira D. A resource guide to understanding cerebral palsy: commentary on collaboration to support health literacy and shared decision making. J Pediatr Rehabil Med. (2021) 14:173–82. 10.3233/PRM-210026 34092662

[19] De Oliveira JM Fernandes RCG Pinto CS Pinheiro PR Ribeiro S de Albuquerque VHC. Novel virtual environment for alternative treatment of children with cerebral palsy. Comput Intell Neurosci. (2016) 2016:8984379, 10 pages. 10.1155/2016/8984379 27403154 PMC4923569

[20] Oktafiolita A Sartinah E Murtadlo M Kunci K. Development of digital peer based pocket book on social behavior of cerebral palsy disability. Jurnal Pendidikan IPS. (2025) 15(1):1–10. 10.37630/jpi.v15i1.2444

[21] Vengatesh G Rajesh R Naveenkumar T. An intelligent computer vision for children affected with cerebral palsy. 2020 International Conference on System, Computation, Automation and Networking (ICSCAN) (2020). p. 1–5. 10.1109/ICSCAN49426.2020.9262275

[22] Islam MM Nooruddin S Karray F Ghulam M. Multi-level feature fusion for multimodal human activity recognition in internet of healthcare things. Inf Fusion. (2023) 94:17–31. 10.1016/j.inffus.2023.01.015

[23] Sorin V Kapelushnik N Hecht I Zloto O Glicksberg BS Bufman HI Integrated visual and text-based analysis of ophthalmology clinical cases using a large language model. Sci Rep. (2025) 15:4999. 10.1038/s41598-025-88948-8 39930078 PMC11811221

[24] Mohsen F Ali H El Hajj N Shah Z. Artificial intelligence-based methods for fusion of electronic health records and imaging data. Sci Rep. (2022) 12(1):17981. 10.1038/s41598-022-22514-4 36289266 PMC9605975

[25] Soenksen LR Ma Y Zeng C Boussioux L Villalobos Carballo K Na L Integrated multimodal artificial intelligence framework for healthcare applications. NPJ Digit Med. (2022) 5(1):149. 10.1038/s41746-022-00689-4 36127417 PMC9489871

[26] Acosta J Falcone G Rajpurkar P Topol E. Multimodal biomedical AI. Nat Med. (2022) 28:1773–84. 10.1038/s41591-022-01981-2 36109635

[27] Lipkova J Chen RJ Chen B Lu MY Barbieri M Shao D Artificial intelligence for multimodal data integration in oncology. Cancer Cell. (2022) 40(10):1095–110. 10.1016/j.ccell.2022.09.012 36220072 PMC10655164

[28] Zhang C Liu S Zhou X Zhou S Tian Y Wang S Examining the role of large language models in orthopedics: systematic review. J Med Internet Res. (2024) 26:e59607. 10.2196/59607 39546795 PMC11607553

[29] Nahar A Paul S Saikia MJ. A systematic review on machine learning approaches in cerebral palsy research. PeerJ. (2024) 12:e18270. 10.7717/peerj.18270 39434788 PMC11493061

