# Supplementary material for: The effectiveness of artificial intelligence models in addressing the concerns of families of children with cerebral palsy: a comparative analysis of ChatGPT, Gemini, and DeepSeek
Source: Front Pediatr. 2025 Dec 8;13:1694035. doi: 10.3389/fped.2025.1694035 (PMC12719435; doi:10.3389/fped.2025.1694035)
Supplement: Supplementary file 1 [file Table1.docx]

**Question 1: What is cerebral palsy and what causes it?**

Cerebral Palsy (CP) is a permanent movement and posture disorder that occurs due to brain damage developing before birth, during birth, or shortly after birth. This disorder is caused by damage in the motor control regions of the developing brain.

**What is Cerebral Palsy?**
Cerebral Palsy (CP) is a non-progressive but permanent neurological disorder that affects a child’s muscle control, movement, posture, and coordination. It occurs due to damage to the motor centers in the brain. CP does not worsen over time, but its effects can last a lifetime.

**Why Does Cerebral Palsy Occur?**
The main causes of CP are divided into three main periods:

**1. Prenatal (Before Birth) Causes:**

- Infections the mother experiences during pregnancy (e.g., rubella, toxoplasmosis)
- Problems affecting brain development during pregnancy
- Brain hemorrhage or blockage of blood vessels in the womb
- Maternal malnutrition or drug/substance use

**2. Perinatal (During Birth) Causes:**

- Difficult and traumatic delivery
- Prolonged lack of oxygen (hypoxia)
- Brain hemorrhage during birth
- Low birth weight and premature birth

**3. Postnatal (After Birth) Causes:**

- Infections such as meningitis or encephalitis
- Brain injuries (fall, accident, etc.)
- Severe jaundice (in the newborn period)
- Brain hemorrhage or oxygen deficiency

**Summary:**
Cerebral Palsy is the result of permanent damage to the developing brain, and these causes may occur before, during, or after birth. The most common causes include oxygen deprivation, infections, and premature birth.

**Question 2: How is cerebral palsy diagnosed? Could it be diagnosed before birth?**

Cerebral Palsy (CP) is usually diagnosed after birth when delays in development or abnormal movements are observed.
It is **not possible** to make a definite diagnosis before birth, but risk factors can be detected.

**🧠 How is Cerebral Palsy Diagnosed?**

**🔍 1. Observation-Based Clinical Evaluation:**

- The doctor checks whether the child achieves motor milestones (holding the head, sitting, walking) at the expected time.
- Muscle tone (very floppy or very stiff), reflexes, balance, and coordination are evaluated.
- Asymmetric movements or involuntary contractions are noted.

**🧪 2. Imaging Methods:**

- Brain MRI (Magnetic Resonance Imaging): Shows the location and type of brain damage.
- CT (Computed Tomography): Used especially in the early period to evaluate brain hemorrhage or structural abnormalities.

**🧠 3. Neurological Examination and Developmental Tests:**

- Neurological examination assesses reflexes, muscle strength, and tone.
- Developmental tests (e.g., Denver II test) assess cognitive and motor skills.

**👶 Could it be Diagnosed Before Birth?**

❌ **No Definite Diagnosis, But Risks Can Be Identified:**
Before birth, it is **not possible** to definitively diagnose CP because the effects of brain damage can only be observed during the child’s developmental process after birth.

⚠️ **Risk Assessment Can Be Done:**

- Serious structural abnormalities in the brain can be detected via prenatal ultrasound.
- If there are known risk factors such as maternal infections or Rh incompatibility, the risk of CP may be high.
- Pregnancy complications (bleeding, premature rupture of membranes, placenta problems) may be warning signs for CP.

**📝 Conclusion:**

- CP is diagnosed through observation, examination, and imaging after birth.
- CP cannot be diagnosed before birth, but high-risk pregnancies can be closely monitored to reduce brain injury risk.

**Question 3: Is there a permanent cure for cerebral palsy?**

No — there is no complete permanent cure for Cerebral Palsy (CP) because CP is the result of **permanent brain damage** that occurs during brain development, and this damage cannot be reversed.
However, effective treatment and rehabilitation methods can be applied to improve the quality of life and independence of individuals with CP.

**🧠 Why Is There No Permanent Cure?**
Cerebral Palsy results from brain damage that is:

- Generally **irreversible**
- **Non-progressive** (it does not worsen over time)
- But symptoms may change over time (depending on the child’s growth)

**🩺 What Are the Treatment Goals?**

- Increase mobility
- Reduce muscle stiffness (spasticity)
- Prevent pain and complications
- Develop independent living skills
- Ensure social and academic integration

**🛠️ Common Treatment and Support Methods:**

1. **Physical Therapy and Rehabilitation**
   - The most essential method in CP treatment
   - Improves balance, muscle strength, flexibility, and walking ability
   - Starting early increases success rates
2. **Speech and Language Therapy**
   - Important for children with swallowing and speech difficulties
3. **Occupational Therapy**
   - Helps develop daily living skills (dressing, eating, etc.)
4. **Medication**
   - Muscle relaxants (e.g., baclofen, botulinum toxin/Botox)
   - Anti-epileptic drugs if epilepsy is present
5. **Surgical Interventions**
   - Orthopedic surgeries to correct deformities caused by muscle shortening
   - Brain or spinal surgery (to reduce spasticity)
6. **Assistive Devices**
   - Walkers, canes, wheelchairs, orthoses

**📌 Summary:**

- There is no permanent cure for CP.
- But with early and multi-faceted interventions, quality of life can be greatly improved.
- Treatment is personalized and can last a lifetime.

**Question 4: When should physiotherapy start in cerebral palsy?**

For a child diagnosed with or suspected of having Cerebral Palsy (CP), physical therapy should begin **as early as possible**.
Since brain development is very rapid in the first years of life, treatment started during this period is **critical** for maximizing the child’s motor functions.

**⏱️ When to Start Physical Therapy?**

✅ **Best Time:** As soon as the first signs appear

- Even if CP is not yet definitively diagnosed, if there is developmental delay, abnormal muscle tone (very floppy or very stiff), or asymmetric movements, physical therapy should begin.
- If there are risk factors (premature birth, difficult delivery, brain hemorrhage), the physical therapist may start a **preventive program**.

**🎯 Benefits of Early Intervention:**

- The brain’s **plasticity** (ability to reorganize) is highest at an early age.
- Prevents musculoskeletal deformities and joint stiffness.
- Improves mobility, self-confidence, and social interaction.
- Sitting, walking, and balance skills are easier to acquire.

**🗓️ Age-Based Recommendations:**

| **Age** | **Intervention Recommendation** |
| --- | --- |
| 0–6 months | Monitor high-risk babies, start therapy if needed |
| 6–12 months | If developmental delays are present, evaluation is essential |
| 12 months+ | If CP symptoms exist, start regular therapy immediately |

**👨‍⚕️ Who Should Refer?**

- Pediatric neurologist or pediatrician
- Developmental pediatric specialist
- Physical medicine and rehabilitation specialist

**📌 Conclusion:**
The earlier therapy starts, the better the child’s potential can be developed. Early and continuous therapy helps prevent or reduce future permanent movement disorders.

**Question 5: Can incorrect exercises harm a child with cerebral palsy?**

Yes — performing the wrong exercises in children with Cerebral Palsy (CP) can cause harm.
Therefore, exercise programs should always be **planned and supervised by specialist physical therapists**.

**❗ Possible Harms of Incorrect Exercises:**

1. **Muscle and Joint Damage**
   - Excessive or inappropriate stretching can cause muscle tears or pain.
   - Incorrect positioning during exercises may lead to dislocations or deformities (e.g., hip dislocation, scoliosis).
2. **Increased Spasticity**
   - Spasticity (muscle stiffness) is common in CP. Wrong techniques may make stiffness worse.
3. **Postural Disorders and Asymmetry**
   - Exercises that ignore muscle balance may cause posture problems (e.g., leaning to one side, curvature).
4. **Pain and Fear of Exercise**
   - If exercises cause pain, the child may avoid them, negatively affecting rehabilitation.
5. **Slowed Progress**
   - Without a personalized plan, exercises may fail to support motor development and could even hinder progress.

**✅ Safe Exercise Practices:**

- The program must suit the child’s age, CP type, and severity.
- Exercises should be planned and supervised by a trained physical therapist.
- Families should participate in the process but should not attempt exercises alone.
- The child should not be forced, and painful movements should be avoided.

**👨‍⚕️ Recommendation:**
Seek help from professionals experienced in special techniques for CP (e.g., Bobath concept, PNF, sensory-motor integration).

**📌 Summary:**
Wrong exercises can indeed harm children with CP.
Therapy should always be carried out by trained professionals, with families involved in a safe and informed way.

**Question 6: Can orthoses be used in cerebral palsy?**

Yes — the use of orthoses in children with Cerebral Palsy (CP) is very common and usually highly beneficial.
Orthoses are supportive devices that help the musculoskeletal system, make movement easier, and prevent deformities.

**🦿 Why Are Orthoses Necessary in CP?**
Common issues in individuals with CP include:

- Muscle stiffness (spasticity)
- Postural problems
- Muscle weakness
- Limited joint mobility
- Balance problems

These issues can lead to permanent deformities (contractures, structural changes) over time. Orthoses help **prevent, correct, or reduce** these problems.

**✅ Benefits of Orthoses in CP:**

- Provide correct alignment of muscles and joints
- Improve walking quality and reduce imbalance
- Prevent contractures and deformities
- Support muscles and reduce energy loss
- Increase independence in daily life

**🔧 Common Types of Orthoses in CP:**

| **Orthosis Type** | **Purpose** |
| --- | --- |
| **AFO (Ankle-Foot Orthosis)** | Corrects foot drop, unstable stepping, and foot deformities |
| **KAFO (Knee-Ankle-Foot Orthosis)** | Provides more support if knee control is weak |
| **SMO (Supramalleolar Orthosis)** | Stabilizes the ankle, provides light support |
| **Hip Abduction Orthosis** | Used in babies to prevent hip dislocation |
| **Knee Braces** | Used at night to prevent knee contractures |

**📌 Important Points:**

- Orthoses should be selected **only** with the recommendation of a specialist doctor and orthopedic technician.
- Incorrect or ill-fitting orthoses can cause pain, pressure, and developmental problems.
- Orthosis use should be combined with physical therapy.

**🧠 Summary:**
Yes — orthoses are often used in CP both preventively and therapeutically.
The type, timing, and method of orthosis use should always be determined by a specialist team, with regular follow-ups.

**Question 7: What is botulinum toxin? Can it be used for my child with cerebral palsy? Are there any side effects?**

Botulinum toxin (commonly known as Botox) is a powerful medication that, when injected into a muscle, causes **temporary relaxation** of that muscle.
In conditions such as Cerebral Palsy (CP), where **muscle stiffness (spasticity)** is present, it can help make movement easier and prevent deformities.

**🧠 What Is Botulinum Toxin?**

- A neurotoxin derived from the bacterium Clostridium botulinum.
- Temporarily blocks nerve signals to the muscle.
- Causes the injected muscle to relax for about **3–6 months**.
- Administered directly into the muscle via injection.

**👶 Can It Be Used for My Child with CP?**
✅ **Yes, in suitable cases.** Botulinum toxin is used in children with CP to:

- Reduce spasticity in the arms or legs
- Improve walking ability
- Prevent the development of contractures (muscle shortening)
- Facilitate orthosis use
- Relax muscles before surgery

**👨‍⚕️ Who Administers It?**

- Pediatric neurologists or physical medicine specialists
- Muscle selection, dosage, and injection sites are usually determined using EMG (electromyography) or ultrasound guidance

**⚠️ Are There Side Effects?**
Generally, it is a safe treatment, but like all medications, it can have side effects.

**Common Side Effects:**

- Mild pain or bruising at the injection site
- Temporary muscle weakness
- Mild fever or restlessness (especially in children)

**Rare but Serious Side Effects:**

- Excessive muscle relaxation
- Temporary swallowing or speech difficulties (if injected into neck/head muscles)
- Allergic reaction (very rare)

**⏳ How Long Does the Effect Last?**

- Usually **3 to 6 months**
- Repeat injections can be given as needed (2–3 times a year)
- Works best when combined with physical therapy

**📌 Summary:**
Botulinum toxin can be safely used in children with CP to reduce muscle stiffness.
Treatment should always be carried out under specialist supervision, using the correct dosage and technique.
Side effects are generally mild and temporary; medical follow-up is important for rare cases.

**Question 8: Is surgery a possible treatment for cerebral palsy? When can surgery be performed?**

In Cerebral Palsy (CP), surgical treatment can be performed in certain special cases.
However, it’s important to note:

🔍 Surgery does **not** completely cure CP, but it can correct certain physical problems, improving the child’s mobility and quality of life.

**🏥 Purposes of Surgery in CP:**

- Correct muscle shortening (contractures)
- Repair joint deformities (e.g., hip dislocation, clubfoot)
- Improve walking ability
- Reduce pain and movement restrictions

**🔧 Main Types of Surgery in CP:**

| **Surgery Type** | **Description** |
| --- | --- |
| **Tendon Lengthening** | Tendons are lengthened to correct muscle shortening (e.g., Achilles tendon) |
| **Muscle Transfer** | A muscle is relocated to change its function |
| **Hip/Spinal Correction Surgery** | Corrects structural problems such as hip dislocation or scoliosis |
| **Selective Dorsal Rhizotomy (SDR)** | Reduces spasticity by cutting certain nerve roots in the spinal cord |

**🕒 When Is Surgery Needed?**

✅ **Indications:**

- When physical therapy and botulinum toxin treatments are insufficient
- When permanent muscle shortening has developed
- When walking and movement are seriously limited
- When pain, pressure sores, or deformities are present
- When bone development is impaired (e.g., hip dislocation has occurred)

**📅 At What Age?**

- Usually considered between **ages 4–8**
- Some orthopedic surgeries can be performed before bone development is complete
- Early surgery can sometimes prevent bigger problems in the future

❗ Surgery decisions should be made **jointly** by the physical therapist, orthopedic surgeon, pediatric neurologist, and the family.

**⚠️ Post-Surgery Rehabilitation Is Essential!**
The success of surgery depends heavily on intensive and regular physical therapy afterward.
The treatment process doesn’t end with surgery — it actually starts again.

**📌 Summary:**
Surgery doesn’t remove CP but can correct some of its effects.
Correct timing, proper technique, and a good rehabilitation plan afterward determine success.

**Question 9: Can my child with cerebral palsy walk normally? When will they be able to walk?**

A child with Cerebral Palsy (CP) may be able to walk depending on the severity and type of their condition, combined with proper treatment and rehabilitation.
The answer to “Can they walk normally?” depends greatly on the **type** and **level** of CP.

**🚶‍♂️ Can My Child with CP Walk?**
✅ Yes — some children with CP can walk independently. This is more likely if:

- CP is mild or moderate
- The child has spastic hemiplegia (only one side of the body affected) — many such children walk independently
- Physical therapy, orthoses, botulinum toxin, and other treatments are used to support mobility

❌ In severe cases (especially spastic quadriplegia or dyskinetic CP):

- Independent walking may not be possible
- Walking aids such as walkers, canes, or wheelchairs may be needed

**🕒 When Can They Walk?**

| **Condition** | **Approximate Walking Age** |
| --- | --- |
| Mild CP | Around 2–3 years old |
| Moderate CP | Between 3–5 years old, often with support |
| Severe CP | Walking may not be possible, or continuous support is needed |

**🧠 Note:**
Children with CP may walk later than their peers, but this does not mean there is no hope.
Progress can vary greatly with patience, specialist support, and proper rehabilitation.

**🔑 Factors Affecting Walking Ability:**

- Type and severity of CP
- Early diagnosis and early start of physical therapy
- Health of muscles and joints (presence of contractures or deformities)
- Family support and motivation
- Correct use of assistive devices
- Neurological and cognitive status (whether there is an accompanying intellectual disability)

**🎯 Interventions That Support Walking:**

- Regular physical therapy and exercise
- Orthosis use (e.g., AFO)
- Botulinum toxin injections
- Surgery (if needed)
- Balance and walking training
- Family education and home support

**📌 Summary:**
Yes — children with CP can walk, but it depends on their medical condition.
In mild cases, independent walking is possible; in more severe cases, walking aids may be required.
Early intervention and consistent rehabilitation greatly increase the chances of walking.

**Question 10: What should I be careful about when dressing my baby with cerebral palsy or changing their diaper?**

When dressing or changing the diaper of a baby with Cerebral Palsy (CP), you must consider factors such as muscle stiffness (spasticity), involuntary movements, and sensitivity.
These daily care tasks can be challenging for a child with CP, but with the right approach, they can be made easier for both the child and the parent.

**👕 Tips for Dressing:**

**✅ 1. Choose Soft and Easy-to-Wear Clothes**

- Prefer zippered or snap-button clothing made of elastic and cotton materials
- Use wide-neck tops and garments with sleeves that are easy to pass through
- Snaps are more practical than buttons

**✅ 2. Move Without Forcing, According to Muscle Tone**

- If spasticity is present, arms or legs may be hard to open — do not force them
- When dressing, put clothes on the affected side first; when undressing, start with the unaffected side
- Before dressing, you can gently stretch or relax the muscles to make movement easier

**✅ 3. Use Supportive Positions During Dressing**

- Dress the baby while they are lying on their side or in a supported sitting position for safety and ease
- Slowly bend arms or legs when passing them through clothing

**🧷 Tips for Diaper Changing:**

**✅ 1. Do Not Lift the Baby Harshly — Roll Them Instead**

- Lifting both legs up together can increase spasticity
- Instead, change the diaper by rolling the baby onto their side
- Slip your hand under the baby’s hips and use a rolling technique to remove and replace the diaper

**✅ 2. Be Gentle During Cleaning**

- Avoid sudden movements and cold contact, as they can increase muscle spasms
- Prefer warm water and a soft cloth instead of wet wipes

**✅ 3. Pay Attention to Skin Health**

- Babies with CP may be more prone to diaper rash and skin irritation due to inactivity
- Keep the diaper area dry and use diaper cream regularly

**🧠 Extra Tips:**

- Speak to your baby in a calm voice during care — this helps reduce anxiety
- If muscle spasms are present, a short massage or relaxation exercise before dressing or changing can help
- Ask your physical therapist for one-on-one guidance on positioning and dressing techniques

**📌 Summary:**
When dressing and changing your baby with CP, it is important to move gently, patiently, and supportively.
Clothing and positions should be chosen according to muscle condition, and forcing should always be avoided.
This will increase your child’s comfort and make care easier for you.
