# Supplementary material for: The effectiveness of artificial intelligence models in addressing the concerns of families of children with cerebral palsy: a comparative analysis of ChatGPT, Gemini, and DeepSeek
Source: Front Pediatr. 2025 Dec 8;13:1694035. doi: 10.3389/fped.2025.1694035 (PMC12719435; doi:10.3389/fped.2025.1694035)
Supplement: Supplementary file 2 [file Table2.docx]

**Question 1: What is cerebral palsy and what causes it?**

**What is Cerebral Palsy (CP)?**

Cerebral Palsy (CP) is a group of neurological disorders that occur as a result of damage to the developing brain before birth, during birth, or in the early postnatal period (usually within the first 2–3 years of life).
It causes **movement and posture disorders**, affects muscle tone, balance, and coordination, but is **non-progressive over time** (the brain damage is permanent, although symptoms may change).

**Why Does Cerebral Palsy Occur?**

The main cause of CP is damage to the **motor control centers** in the brain.
This damage can occur in the following situations:

**1. Prenatal (During Pregnancy) Causes:**

- Abnormalities in brain development (genetic mutations or structural brain malformations)
- Maternal infections: toxoplasmosis, rubella, cytomegalovirus (CMV), syphilis
- Mother’s chronic illnesses: uncontrolled diabetes, thyroid disorders
- Placental problems or nutritional deficiencies
- Maternal use of alcohol, cigarettes, or drugs
- Risk of premature birth (increases the risk of brain hemorrhage in premature babies)

**2. Perinatal (During Birth) Causes:**

- Oxygen deficiency (prolonged or difficult labor, umbilical cord problems)
- Traumatic birth (brain hemorrhage, skull injury)
- Severe jaundice (kernicterus: bilirubin damaging the brain)

**3. Postnatal (After Birth) Causes:**

- Brain infections: meningitis, encephalitis
- Head trauma (severe falls, accidents)
- Stroke or brain hemorrhage (especially in premature babies)
- Severe hypoglycemia (low blood sugar)

**Important Notes:**

- CP is **not contagious** and is generally **not hereditary** (in most cases).
- **Early intervention** (physical therapy, special education) can reduce symptoms.
- People with CP may have normal intelligence; physical disability does **not** necessarily mean intellectual disability.

**Question 2: How is cerebral palsy diagnosed? Could it be diagnosed before birth?**

**How is CP Diagnosed?**

Cerebral Palsy is usually suspected **months or years after birth** when delays or abnormalities in a child’s motor development are noticed.
A definite diagnosis is made using **clinical examination, imaging methods, and neurological evaluation**.

**Methods Used in the Diagnosis Process:**

**Clinical Examination:**

- Delay in motor skills (inability to hold the head up, sit, crawl)
- Abnormal muscle tone (excessive stiffness [spasticity] or floppiness [hypotonia])
- Checking reflexes (overactive reflexes, such as the Babinski reflex)

**Imaging Tests:**

- **Brain MRI** (most detailed method): Shows brain damage, lesions, or developmental abnormalities.
- **Cranial ultrasound** (in premature babies): Detects bleeding or brain tissue problems.
- **CT scan (Computed Tomography):** Used when MRI is not possible.

**Additional Tests:**

- **EEG** (if seizures are suspected)
- **Genetic testing** (rarely, to rule out other syndromes)

**Is Prenatal (Before Birth) Diagnosis Possible?**

No — Cerebral Palsy **cannot** be definitively diagnosed before birth.

**Reasons:**

- CP is caused by damage to the brain’s motor regions, which most often occurs during birth or after birth.
- Prenatal ultrasound or fetal MRI can detect some **structural brain abnormalities** (e.g., severe brain malformations), but these are not definitive indicators of CP.
- Risk factors (premature birth, maternal infections) can be detected, but it is **not possible** to say, “This baby will have CP.”

**However:**

- **High-risk pregnancies** (e.g., maternal infection, placental problems) can be closely monitored to prevent premature birth.
- In the early postnatal period (especially in premature babies), if brain damage is detected via MRI or ultrasound, the risk of CP can be predicted.

**Why is Early Diagnosis Important?**

- The earlier physical therapy, special education, and rehabilitation begin, the less functional loss the child will experience.
- From as early as 3–6 months, if there are suspicious signs (e.g., using one side of the body less), a pediatric neurologist should be consulted.

**Summary:**

- CP is diagnosed **after birth**; a definitive prenatal diagnosis is not possible.
- Early intervention can improve quality of life.

**Question 3: Is there a permanent cure for cerebral palsy?**

**Short answer:** No — there is no completely curative treatment for Cerebral Palsy (CP) because brain damage is permanent.
However, with **early and continuous treatment**, symptoms can be reduced, mobility can be improved, and quality of life can be enhanced.

**Treatment Methods and Goals**

CP treatment requires a **multidisciplinary approach** (neurologist, physical therapist, orthopedist, speech therapist, etc.).

**1. Physical Therapy and Rehabilitation**
**Purpose:** Strengthen muscles, maintain joint range of motion, improve balance and walking skills.
**Methods:**

- Special techniques such as Bobath therapy, Vojta therapy
- Hydrotherapy (water-based exercises)
- Robotic walking devices (Lokomat)

**2. Medication**
To reduce muscle spasticity:

- Oral medications (Baclofen, Diazepam)
- Botox injections (to relax specific muscles)

For seizure control (if epilepsy is present).

**3. Surgical Interventions**

- Orthopedic surgeries: Muscle release (tendon lengthening), bone correction (scoliosis, hip dislocation)
- Baclofen pump: Administers medication into the spinal fluid for severe spasticity

**4. Assistive Devices**

- Walking aids (walker, crutches)
- Special orthoses (AFO: ankle-foot orthoses)
- Electric wheelchairs (in severe cases)

**5. Speech and Swallowing Therapy**

- Strengthen tongue and oral muscles, improve communication skills
- Alternative communication methods (tablet apps, sign language)

**6. Alternative Therapies**

- Hippotherapy (horseback riding therapy)
- Acupuncture (for pain management)

**New Promising Developments**

- **Stem cell therapy:** Still experimental, effectiveness unproven
- **Therapies that enhance neuroplasticity:** Research on methods that support the brain’s ability to reorganize itself

**Important Points**

✔ Treatment is **individualized** — every person with CP has different needs
✔ Early intervention (especially ages 0–3) is the most critical period
✔ Regular follow-up and rehabilitation may be lifelong

**Conclusion:**
While no treatment can completely eliminate CP, the right support can increase independence and reduce pain. 💪

**Question 4: When should physiotherapy start in cerebral palsy?**

**Answer:** As early as possible! Ideally, physical therapy should start **as soon as the diagnosis is made** (or even at the suspicion stage).

**1. Importance of Starting Early (Critical Period: Under 3 Years Old)**

- In this period, brain **plasticity** (flexibility) is at its highest — physical therapy can strengthen neural connections.
- Early intervention, while the musculoskeletal system is still forming, can **prevent contractures** (muscle shortening) and deformities.
- Delayed motor skills (head control, sitting, crawling) can be supported more quickly.

**2. Scenarios Based on Starting Time**

- **Premature/High-Risk Babies:** Start immediately after birth (even in the neonatal intensive care unit, passive movements can be initiated).
- **Mild–Moderate CP Symptoms:** Begin between 3–6 months (e.g., inability to hold head, asymmetric movement).
- **Severe CP:** Start as soon as diagnosed (early intervention to reduce spasticity).

**3. Types of Physical Therapy and Applications by Age**

| **Age Range** | **Therapy Goals** | **Methods** |
| --- | --- | --- |
| **0–12 months** | Head control, hand-eye coordination, rolling, sitting | Bobath, passive-active exercises, sensory-motor stimulation |
| **1–3 years** | Crawling, standing, balance, pre-walking skills | Vojta therapy, hydrotherapy, balance boards |
| **3+ years** | Walking training, fine motor skills (grasping, writing), daily living activities | Robotic rehabilitation, orthosis-assisted exercises |

**4. What Happens If Therapy Starts Late?**

- Muscle stiffness (spasticity) and **joint deformities** (scoliosis, hip dislocation) progress rapidly.
- Functional losses (inability to walk, use hands) may become permanent.
- Psychosocial development is negatively affected (loss of self-confidence, social withdrawal).

**Key Advice for Families**

✔ Do **not** say: “Let’s wait, maybe it will improve.” Every month lost in the early period makes recovery harder.
✔ Support therapy with **home exercise programs** (do the movements shown by the therapist daily).
✔ Play-based activities (rolling a ball, reaching for colorful toys) should be part of treatment.

**Summary:**

- Starting physical therapy early is the most effective treatment in CP.
- Ideal time: at diagnosis (or preventively if risk factors are present).
- For those who say, “We are late”: It’s never too late, but the earlier, the better the results! 💙

**Question 5: Can incorrect exercises harm a child with cerebral palsy?**

**Absolutely YES!**
Unsupervised or incorrect exercises can increase muscle spasticity, cause permanent joint damage (contractures, dislocations), and lead to pain in the child.

**Risks of Wrong Exercises**

**1. May Increase Spasticity**

- For example, **overstretching spastic muscles** (forcing tiptoe position like a ballerina) can make the muscle contract even more.
- Sudden, uncontrolled movements (rapid pulling of arms or legs) can trigger a spastic response.

**2. May Cause Joint and Bone Deformities**

- **Hip dislocation risk:** Forcing the legs apart, especially in children with high spasticity, can worsen hip dysplasia.
- **Scoliosis (spinal curvature):** Sitting or balance exercises done in the wrong position can make the condition worse.

**3. Pain and Psychological Trauma**

- Forceful exercises create **fear of pain** in the child, leading to resistance to physical therapy.
- Loss of motivation: Harsh, non-enjoyable exercises can make the child refuse treatment.

**Examples of Risky Exercises**

- Unsupervised stretching movements (especially bending forward with straight knees)
- Resistance exercises with weights (increase tone in spastic muscles)
- Complex movements the child is not ready for (e.g., forced treadmill walking)

**Correct Approach**

**Must Be Supervised by a Specialist:**

- A physical therapist should prepare an **individualized program**.
- Use **neurological physiotherapy techniques** (Bobath, Vojta).

**Pain-Free and Gradual Adaptation:**

- Combine exercises with play (throwing a ball, catching bubbles).
- Stretch slowly and in a controlled way.

**Caution for Home Exercises:**

- Families should repeat only the simple, safe movements shown by the therapist.
- Never attempt random stretching or loading.

**Example: Safe vs. Risky Exercises**

| **Safe Exercise** | **Risky Exercise** | **Why Risky?** |
| --- | --- | --- |
| Cycling legs while lying on the back | Rapidly stretching the legs | Triggers spasticity |
| Leaning forward while sitting with knees bent | Forcing forward bend with straight knees | Overstretches hamstrings |
| Grasping a soft ball | Lifting heavy dumbbells | Increases contraction in spastic hand |

**Golden Rules for Families**

✔ The idea that “If I push harder, it will get better” is **WRONG**!
✔ If the child cries, stop — pain means the exercise is wrong.
✔ Keep constant communication with the physical therapist, regularly update the exercise plan.

**Reminder:** CP rehabilitation is like a marathon. Being patient, informed, and consistent gives the best results. 🌟

**Question 6: Can orthoses be used in cerebral palsy?**

**Answer:** Yes — orthoses are frequently used in children with Cerebral Palsy (CP) and make a significant contribution to functional improvement.
However, correct selection and timing of use are critically important.

**1. Main Purposes of Orthoses in CP**

- Keep joints and muscles in the correct position (prevent deformities)
- Support walking ability (enable balanced stepping)
- Delay muscle shortening (contractures) caused by spasticity
- Facilitate daily living activities (standing, sitting)

**2. Most Common Types of Orthoses**

**A) AFO (Ankle–Foot Orthosis)**
**Function:**

- Holds the ankle in a neutral position
- Corrects gait (prevents toe-walking)
- Improves balance
  **Used for:**
- Children with spastic diplegia (mainly lower limb involvement)
- Shortened Achilles tendon

**B) KAFO (Knee–Ankle–Foot Orthosis)**
**Function:**

- Stabilizes both the knee and ankle at the same time
- Aligns legs properly in severe spasticity
  **Used for:**
- Quadriplegic CP with poor knee control

**C) Spinal Orthoses (Scoliosis Braces)**
**Function:**

- Slows the progression of spinal curvature (scoliosis)
  **Used for:**
- Children with poor sitting balance and weak trunk control

**D) Wrist–Hand Orthoses**
**Function:**

- Maintains the hand in a functional position
- Supports grasp ability

**3. Factors to Consider in Orthosis Selection**

- Type of CP and muscle tone (spastic, hypotonic, dystonic)
- Age and growth rate (orthoses must be replaced more often during growth periods)
- Functional goals (walking vs. sitting balance)

**Example:**

- Child with walking potential → **Dynamic AFO** (flexible material)
- Severe quadriplegic CP → **KAFO + special seating support**

**4. Risks of Improper Orthosis Use**

- Skin sores (especially in children with sensory loss)
- Muscle weakness (with overly prolonged use)
- Psychological resistance (child may find it uncomfortable and want to remove it)

**Solution:**

- Introduce orthoses gradually (start with 1–2 hours/day)
- Regularly check for redness around the ankle

**5. Orthosis + Physical Therapy Cooperation**

- Orthoses **are not sufficient on their own** — must be combined with physical therapy.
- Example: A child wearing an AFO should also do walking training.

**6. Practical Advice for Families**

✔ Do not make the child wear orthoses “all the time” — follow the therapist’s recommended duration (usually 2–4 hours/day to start).
✔ Pay attention to discomfort — if the child cries or complains, check the orthosis.
✔ Replace as the child grows — have orthosis fit checked every 6 months.

**Summary:**

- Orthoses add functional benefits in CP, but must be selected correctly.
- The most common is the AFO.
- They should always be used together with physical therapy.

**Question 7: What is botulinum toxin? Can it be used for my child with cerebral palsy? Are there any side effects?**

**What is Botulinum Toxin (Botox)?**

Botulinum toxin is a **neurotoxin** that, in controlled doses, is used in medicine for the treatment of spasticity.
When injected into a muscle, it **temporarily blocks nerve–muscle communication**, reducing excessive muscle contraction (spasticity).

**Use in Children with CP**

**1. When is it Used?**

- Muscle shortening due to spasticity (e.g., calf muscle tightness, toe-walking)
- Increasing joint range of motion (as support for physical therapy)
- Reducing painful muscle spasms
- As a temporary solution before surgery (to delay surgery timing)

**2. Who is it Suitable For?**

- Children aged **2 years and older** (safety data available for this age group)
- Those with **focal spasticity** (only certain muscle groups are affected)
- Families who are committed to continuing physical therapy (Botox alone is not enough)

**3. Who is it NOT Suitable For?**

- Those with widespread muscle involvement (whole-body spasticity)
- Those with severe muscle weakness or significant bone deformity
- Those allergic to botulinum toxin

**Side Effects and Risks**

**Mild (Temporary) Side Effects:**

- Pain/redness at the injection site
- Temporary muscle weakness (may last a few weeks)
- Mild flu-like symptoms (fever, fatigue)

**Serious (Rare) Side Effects:**

- Swallowing or breathing difficulty (if injected into neck muscles)
- Excessive muscle weakness (if incorrect dose is used)
- Urinary incontinence (if pelvic floor muscles are affected)

**Note:** Side effects usually disappear within **2–3 weeks**.

**Procedure**

1. **Assessment:** Pediatric neurologist and physical therapist evaluate spasticity
2. **Injection:** Injected directly into the muscle using special needles under ultrasound guidance (1–2 minutes)
3. **After Injection:**
   - No heavy exercise for 48 hours
   - Physical therapy is intensified after 1 week
4. **Effect Duration:** 3–6 months (may require repetition)

**Important Questions for Families**

**“Will my child benefit from Botox?”**

- Answer: It is effective if spasticity is limited to certain muscles and used in the early period (before contractures develop).

**“Is it painful?”**

- Answer: There is needle pain, but it can be reduced with topical anesthetic creams or mild sedation.

**“What if physical therapy is not continued?”**

- Answer: Since Botox’s effect is temporary, muscles will stiffen again without therapy. Physical therapy ensures permanent functional gains.

**Alternatives and Advantages of Botox**

| **Treatment** | **Advantages** | **Disadvantages** |
| --- | --- | --- |
| **Botulinum Toxin** | Non-surgical, quick effect (3–7 days) | Temporary (3–6 months) |
| **Oral Medication** | Affects the whole body | Can cause drowsiness, weakness |
| **Surgery** | Permanent solution | Risky, long rehabilitation |

**Summary:**
✅ Botox can be safely used in children with CP, but only under specialist supervision.
✅ Best results occur when **combined with physical therapy**.
✅ Side effects are generally mild and temporary.

**Question 8: Is surgery a possible treatment for cerebral palsy? When can surgery be performed?**

**Answer:** Yes — in some children with CP, surgery is an important treatment option, but it is not suitable for every patient.
The decision for surgery depends on the child’s age, CP type, musculoskeletal problems, and functional potential.

**1. When is Surgery Needed?**

**A) Muscle–Tendon Surgeries**

- **Achilles tendon lengthening:** If there is toe-walking
- **Hamstring lengthening:** If knees remain bent while walking
- **Adductor tendon release:** If legs are crossing (scissoring)

**B) Bone Correction Surgeries**

- **Hip dislocation surgery** (femoral osteotomy): If the hip joint has dislocated
- **Foot deformities** (pes equinus): To improve balance
- **Scoliosis surgery** (spinal fusion): If there is severe curvature

**C) Selective Dorsal Rhizotomy (SDR)**

- Used only in **spastic diplegia** (excessive spasticity in the legs)
- Reduces spasticity by cutting overactive nerves from the spinal cord

**2. Appropriate Timing for Surgery**

**A) Early Surgery (Ages 5–8)**

- For mild–moderate deformities in children with walking potential
- Examples: Achilles tendon lengthening, hamstring release

**B) Late Surgery (Over Age 10)**

- For bone deformities (hip dislocation, scoliosis) — usually delayed until growth slows
- Goal: Reduce pain, improve sitting/lying position

**C) Groups That Should Never Have Surgery**

- Severe quadriplegic CP (whole-body involvement) with advanced muscle weakness
- Children with uncontrolled seizures (high anesthesia risk)

**3. Advantages and Risks of Surgery**

| **Advantages** | **Risks** |
| --- | --- |
| Reduces pain | Infection, bleeding |
| Improves walking ability | Excessive muscle looseness (weakness) |
| Improves sitting/lying comfort | May require repeat surgery |
| Permanently reduces spasticity (SDR) | Psychological trauma |

**4. Post-Surgery Process**

- **1–2 weeks:** Use of cast/splint (depending on surgery type)
- **6 weeks–3 months:** Intensive physical therapy (to retrain muscles)
- **6 months later:** Final results become clearer

**Important:** Surgery alone does **not** cure CP — must be supported by rehabilitation.

**5. How is the Surgery Decision Made?**

- Joint evaluation by pediatric neurologist, orthopedic surgeon, and physical therapist
- Tests such as **EMG** and **gait analysis** may be used
- Discussion of family expectations (e.g., “Will my child be able to walk?”)

**Key Warnings for Families**

⚠ Do **not** think “Surgery will fix everything” — in CP, surgery aims to **improve quality of life**, not perform miracles.
⚠ Avoid early surgery (before age 5 — bones are not fully developed).
⚠ Selective Dorsal Rhizotomy (SDR) requires strict patient selection.

**Summary:**

- Surgery in CP is especially beneficial for children with walking potential.
- The most common procedures are tendon release, bone correction, and SDR.
- Without correct timing and proper rehabilitation, surgery can fail.

**Question 9: Can my child with cerebral palsy walk normally? When will they be able to walk?**

**Answer:**
The walking potential of a child with Cerebral Palsy (CP) depends on **the type of CP, the severity of muscle involvement, and early intervention programs**.
Some children can walk independently, while others may need walkers or wheelchairs.

**1. CP Types and Walking Potential**

**A) Spastic Diplegia (Mainly Leg Involvement)**

- Highest walking potential group
- **70–80%** can walk independently or with a walker
- Walking age: Usually **2–5 years** (later than average)

**B) Mild Spastic Hemiplegia (One-Sided Involvement)**

- Almost all can walk independently
- Walking age: **18 months–3 years** (may have slight limp)

**C) Spastic Quadriplegia (Whole-Body Involvement)**

- Low walking probability (**10–20%**)
- Most need wheelchair or standing support devices

**D) Dystonic/Athetoid CP**

- Walking is difficult due to balance problems

**2. Factors Affecting Walking Ability**

✔ Severity of muscle spasticity (and how well it can be reduced)
✔ Presence of joint deformities (hip dislocation, foot drop)
✔ Balance and coordination skills
✔ Whether early and regular physical therapy is given
✔ Family support and motivation

**3. When Does Walking Potential Become Clear?**

- **2–3 years:** Children with mild CP start walking
- **5–6 years:** Moderate CP cases may take steps with a walker
- **After age 7:** If a child still cannot walk, independent walking becomes less likely

**Note:** Children with CP can walk **1–3 years later** than their peers.

**4. How to Support Walking?**

**A) Physical Therapy**

- Techniques like Bobath and Vojta for balance and stepping
- Treadmill training

**B) Orthoses and Assistive Devices**

- AFO (ankle–foot orthosis) to improve balance
- Walkers or crutches

**C) Surgical Intervention (If Needed)**

- Achilles tendon lengthening, adductor release

**D) Home Exercises**

- Reaching for toys, crawling, taking supported steps holding onto furniture

**5. Realistic Expectations**

- **Mild CP:** Can walk normally (possible mild limp)
- **Moderate CP:** May walk with a walker
- **Severe CP:** Wheelchair may be necessary

**Important:**

- “Normal walking” is defined differently for each child
- Every child’s development pace is unique — patience is key

**6. Advice for Families**

✔ Do not compare your child to others
✔ Never skip physical therapy
✔ Reward walking efforts (motivation through games)
✔ Stay hopeful and celebrate small progress

**Summary:**
Many children with CP can be independent in life with the right support.
Walking is **not** the only measure of success! 💙

**Question 10: What should I be careful about when dressing my baby with cerebral palsy or changing their diaper?**

When dressing or changing the diaper of a baby with cerebral palsy (CP), it's important to be gentle, patient, and attentive to their unique needs. Here are key things to be cautious about:

### ****1. Positioning & Support****

- **Avoid sudden movements** – Babies with CP may have stiff or floppy muscles, so handle them slowly and support their head, neck, and limbs.
- **Use supportive surfaces** – Change diapers on a soft, padded surface to prevent discomfort. A slightly elevated changing table (with safety straps) can help if they have reflux.
- **Side-lying or bent-leg position** – If your baby has high muscle tone (spasticity), gently bend their hips and knees to ease diaper changes.

### ****2. Clothing Choices****

- **Opt for adaptive clothing** – Choose garments with wide neck openings, snaps, or Velcro instead of buttons or tight pullovers.
- **Avoid restrictive fabrics** – Stiff or tight clothing can irritate sensitive skin or worsen muscle stiffness.
- **Layer for temperature control** – Some babies with CP struggle with body temperature regulation.

### ****3. Diapering Tips****

- **Check for discomfort signs** – Babies with CP may not communicate pain clearly, so watch for grimacing, crying, or stiffening.
- **Use hypoallergenic wipes/diapers** – Many children with CP have sensitive skin or allergies.
- **Prevent diaper rash** – Change frequently, apply barrier cream, and ensure the area is dry.

### ****4. Handling Spasticity or Floppiness****

- **For stiff limbs (spasticity):** Gently rotate the hip inward when lifting legs to avoid triggering stiffness.
- **For low muscle tone (hypotonia):** Fully support their body to prevent sudden slips.

### ****5. Hygiene & Safety****

- **Watch for reflux/spitting up** – Keep the baby’s head slightly elevated after feeding before changing.
- **Never leave unattended** – Even if they don’t roll over typically, involuntary movements can pose risks.

### ****6. Emotional Comfort****

- **Stay calm & reassuring** – Some babies with CP may startle easily. Use a soothing voice and avoid abrupt noises.
- **Incorporate therapy exercises** – If recommended by a physiotherapist, gentle stretches during dressing can help.

Would you like recommendations for specific adaptive clothing brands or diaper-changing tools? I’m happy to help further!
