# Supplementary material for: The effectiveness of artificial intelligence models in addressing the concerns of families of children with cerebral palsy: a comparative analysis of ChatGPT, Gemini, and DeepSeek
Source: Front Pediatr. 2025 Dec 8;13:1694035. doi: 10.3389/fped.2025.1694035 (PMC12719435; doi:10.3389/fped.2025.1694035)
Supplement: Supplementary file 3 [file Table3.docx]

**Question 1: What is cerebral palsy and what causes it?**

**What is Cerebral Palsy (CP)?**

Cerebral Palsy (CP) is a permanent neurological condition that affects communication between the brain and muscles, causing movement and posture disorders.
It results from brain damage or developmental problems in the brain and is usually diagnosed during childhood.
Individuals with CP may experience muscle weakness, involuntary movements, balance issues, and lack of coordination.
The severity of the condition varies from person to person — some may have mild symptoms, while others may face more serious physical limitations.

**Why Does Cerebral Palsy Occur?**

The factors that cause cerebral palsy are varied and usually arise from developmental problems or damage in the brain.
Although the exact cause is not always identifiable, risk factors are generally present **before birth, during birth, or after birth**.

**Prenatal Causes (During Pregnancy):**

- **Brain Development Abnormalities:** Improper development of the brain, which may be linked to genetic factors or environmental influences, can lead to CP.
- **Infections:** Maternal infections during pregnancy — such as rubella, toxoplasmosis, cytomegalovirus (CMV), or Zika virus — can damage the developing baby’s brain.
- **Rh Incompatibility:** Blood type incompatibility between the mother and baby can cause severe jaundice in the fetus, which can lead to brain damage.
- **Exposure to Toxins:** Maternal exposure to alcohol, cigarettes, or certain drugs during pregnancy can adversely affect fetal brain development.
- **Metabolic Disorders:** Uncontrolled maternal conditions such as diabetes or thyroid disease can impact fetal brain development.

**Perinatal Causes (During Birth):**

- **Oxygen Deprivation (Asphyxia):** Inadequate oxygen supply to the baby’s brain during delivery can damage brain cells. This may occur during prolonged or difficult labor.
- **Premature Birth:** Premature babies have underdeveloped brains, making them more vulnerable to injury. The risk of brain hemorrhage or white matter injury (periventricular leukomalacia) is increased.
- **Low Birth Weight:** Often associated with prematurity, low birth weight further increases the risk of CP.

**Postnatal Causes (First Years After Birth):**

- **Brain Infections:** Serious infections such as meningitis (inflammation of the brain membranes) or encephalitis (inflammation of the brain tissue) can cause permanent brain damage.
- **Head Trauma:** Serious head injuries during infancy or early childhood can lead to brain damage and CP.
- **Severe Jaundice:** Untreated severe newborn jaundice can cause kernicterus, a condition in which excess bilirubin damages the brain.
- **Drowning or Poisoning:** Events that cause oxygen deprivation or certain poisonings can also increase the risk of brain injury.

**Note:** CP often results from the combination of multiple risk factors, and each case is unique.
Therefore, **early diagnosis and a multidisciplinary treatment approach** are crucial to improving quality of life for individuals with CP.

**Question 2: How is cerebral palsy diagnosed? Could it be diagnosed before birth?**

**How is CP Diagnosed?**

Cerebral Palsy (CP) is usually diagnosed **during infancy or early childhood**, as symptoms become more apparent during the child’s growth and development.
The diagnostic process is typically carried out by a **pediatric neurologist** or a **developmental pediatrician** through a comprehensive evaluation, which includes:

**1. Detailed Medical History:**

- The doctor will review the child’s birth history (premature birth, difficult labor, oxygen deprivation, etc.).
- Developmental milestones such as head control, rolling over, sitting, crawling, and walking are examined for delays.
- Possible risk factors (infections, injuries, etc.) are noted.
- Family history of similar conditions is investigated.

**2. Physical and Neurological Examination:**

- Assessment of muscle tone (tightness or floppiness)
- Reflex evaluation (persistence of primitive reflexes)
- Observation of posture, balance, and coordination
- Identification of CP-specific symptoms such as:
  - **Spasticity** (muscle stiffness)
  - **Ataxia** (coordination disorder)
  - **Athetosis** (involuntary movements)
- Evaluation of developmental delays and motor asymmetries

**3. Imaging Tests:**

- **Magnetic Resonance Imaging (MRI):** Provides detailed 3D images of the brain and shows areas of injury or abnormal development. Most common and effective imaging method for CP diagnosis.
- **Cranial Ultrasound (in infants):** Quick and practical, often used in premature babies to detect brain hemorrhage or abnormalities (less detailed than MRI).
- **Computed Tomography (CT):** Rarely used, provides less detail than MRI and involves radiation exposure.

**4. Other Tests:**

- **Electroencephalogram (EEG):** Used for children with a history of seizures to assess brain electrical activity.
- **Laboratory Tests:** Rarely performed; may include blood, urine, or skin tests to rule out genetic or metabolic disorders.

**Follow-Up in the Diagnostic Process**

In some cases — especially in very young infants or those with mild symptoms — a definitive diagnosis may not be made immediately.
Doctors may follow the child’s development over time to determine whether symptoms persist and meet the criteria for CP.

**Could CP Be Diagnosed Before Birth?**

Generally, **CP cannot be definitively diagnosed before birth** for several reasons:

1. **Visibility of Brain Damage:**
   - The brain damage or developmental issues causing CP may not be easily detected during routine prenatal ultrasounds.
   - Brain development is complex, and minor injuries or functional impairments may only become evident after birth through motor delays.
2. **Timing of Symptom Onset:**
   - CP is a movement and posture disorder, so symptoms usually become apparent only when the baby starts moving and developing motor skills.
   - Even if damage occurs before birth, its effects are often not noticed until months or years later.
3. **Variety of Causes:**
   - CP can arise before, during, or after birth.
   - Events like oxygen deprivation during delivery cannot be detected before birth.

**When Can Risks Be Identified Prenatally?**

- Certain **risk factors** during pregnancy — such as maternal infections (rubella, toxoplasmosis), severe bleeding, placental problems, or genetic syndromes — can indicate that the baby is at higher risk for CP.
- **Fetal MRI** may be performed in rare cases when serious brain abnormalities are suspected. This helps detect structural brain issues but **does not directly diagnose CP**, as not all CP cases involve visible structural abnormalities.

**Summary:**

- CP is usually diagnosed after birth when delays in motor development and abnormal movement patterns are observed.
- In the prenatal period, only **risk factors** can be identified — a definitive diagnosis is rarely possible.

**Question 3: Is there a permanent cure for cerebral palsy?**

Unfortunately, there is **no definitive and permanent cure** for Cerebral Palsy (CP).
The brain damage that causes CP is permanent and **cannot be fully repaired or reversed** with current medical methods.
However, treatment and rehabilitation programs aim to **significantly improve quality of life**, manage symptoms, develop functional skills, and help individuals reach their full potential.

**Treatment Approaches and Goals**

A **multidisciplinary approach** is essential in CP treatment.
This means doctors and therapists from different specialties work together to create a personalized plan based on the patient’s individual needs.

**Main goals of treatment include:**

- **Increasing functional independence:** Helping with daily activities such as eating, dressing, and personal care.
- **Improving motor skills:** Enhancing balance, coordination, muscle strength, and range of motion.
- **Preventing or reducing complications:** Managing muscle contractures, joint deformities, pain, and other secondary issues.
- **Improving quality of life:** Supporting social participation, communication, and overall well-being.

**Treatment Methods**

**1. Physical Therapy (Physiotherapy)**

- Core component of CP treatment
- Aims to regulate muscle tone, increase strength, improve flexibility, balance, and coordination
- Includes individualized exercise programs, stretching, and the use of assistive devices (orthoses, walkers, etc.)

**2. Occupational Therapy**

- Focuses on increasing independence in daily activities (dressing, feeding, writing)
- Improves hand–eye coordination, fine motor skills, and adaptive equipment use

**3. Speech and Swallowing Therapy**

- Addresses speech difficulties (dysarthria), swallowing problems (dysphagia), and communication skills
- Includes strengthening exercises for speech muscles, alternative communication methods (picture cards, communication devices), and safe swallowing techniques

**4. Medication**

- **Muscle relaxants** (e.g., Baclofen, Diazepam) taken orally or delivered directly into the spinal fluid via an intrathecal baclofen pump
- **Botulinum toxin injections** to temporarily reduce spasticity in specific muscles and improve the effectiveness of physiotherapy
- **Anticonvulsants** for seizure control in patients with epilepsy

**5. Surgical Treatments**

- To release tight muscles, lengthen tendons, correct bone deformities, or reduce severe spasticity
- **Selective Dorsal Rhizotomy (SDR):** Cutting certain nerve fibers in the spinal cord to permanently reduce spasticity
- **Orthopedic surgeries:** Correcting contractures or bone deformities

**6. Special Education and Psychological Support**

- Enhances academic and social development in children with CP
- Counseling helps both the individual and their family cope with the condition

**7. Stem Cell Therapy and Gene Therapy (Experimental)**

- Stem cell therapy aims to repair damaged brain cells or create new ones — still under research and not yet part of routine treatment
- Gene therapy may be a future option for genetically linked CP, but remains in the research phase

**Summary:**
CP is a permanent condition, but with **early diagnosis** and a **comprehensive, continuous treatment and rehabilitation program**, quality of life can be greatly improved, and individuals can reach their maximum potential.

**Question 4: When should physiotherapy start in cerebral palsy?**

In Cerebral Palsy (CP), starting physical therapy **as early as possible — even immediately after diagnosis — is extremely important**.
Experts emphasize that early intervention has highly positive effects on a child’s development and quality of life.

**Reasons Why Early Intervention is Important**

**1. Neuroplasticity:**

- The brain’s ability to learn and adapt (neuroplasticity) is highest in early life.
- Starting physical therapy in this period provides greater opportunities for the brain to compensate for damaged areas and form new neural connections.

**2. Motor Skill Development:**

- Early therapy can speed up the development of motor skills such as head control, sitting, crawling, and walking.
- It can also significantly improve muscle control, balance, and flexibility.

**3. Prevention of Secondary Complications:**

- Helps prevent or slow the progression of secondary problems linked to CP, such as muscle contractures, joint deformities, and postural disorders.

**4. Independence:**

- Early intervention contributes to greater independence in daily activities and improves overall quality of life.

**Key Recommendation from Studies**

Research shows that the time between recognizing motor development delay in children with CP and starting regular physical therapy is a **lost opportunity**.
For this reason, high-risk infants or children showing developmental delay should be referred to specialists (pediatric neurologists, physical medicine and rehabilitation doctors) **without delay** to start therapy promptly.

**Summary:**
Starting physical therapy early in CP maximizes developmental potential, prevents complications, and supports independence.
Delays in beginning treatment can limit outcomes, so early referral is critical.

**Question 5: Can incorrect exercises harm a child with cerebral palsy?**

**Yes — wrong exercises in CP can definitely harm the child.**
Although the goal of physiotherapy is to improve muscle strength, flexibility, and motor skills, using incorrect techniques or working without professional supervision can cause serious problems.

**Possible Harms of Incorrect Exercises**

**1. Worsening Existing Damage:**

- The muscles and joints of children with CP can be sensitive.
- Wrong stretching or forcing movements can worsen existing muscle contractures or joint deformities — or even cause new injuries.

**2. Pain and Discomfort:**

- Doing more than the child can handle, or using inappropriate methods, can cause significant pain.
- This may make the child unwilling to participate in therapy and reduce cooperation.

**3. Joint Injuries:**

- Uncontrolled or overly forceful movements can damage joints, causing sprains, dislocations, or joint inflammation.
- This is especially risky when excessive force is applied to spastic muscles.

**4. Muscle Fatigue and Exhaustion:**

- Exercise programs beyond the child’s physical capacity can cause extreme fatigue.
- This reduces the child’s ability to perform daily activities and slows recovery.

**5. Increased Postural Problems:**

- Incorrect exercises can worsen body imbalance or strengthen the wrong muscle groups, leading to worsening of existing postural problems.
- May contribute to the progression of scoliosis (spinal curvature).

**6. Psychological Effects:**

- Constant pain, strain, and a sense of failure can lower the child’s morale and self-confidence, leading to a negative attitude toward therapy.

**Conclusion:**

For these reasons, **exercise programs in CP must be designed and supervised by a qualified physical therapist**, tailored to the child’s age, physical condition, and type of CP.
Even at home, families should only perform exercises taught by the therapist and under their guidance.
“Alternative” exercise methods from untrained individuals should be avoided.

**Safe and effective CP rehabilitation always requires professional guidance.**

**Question 6: Can orthoses be used in cerebral palsy?**

**Yes — orthosis use is very common and an important treatment method in Cerebral Palsy (CP).**
Orthoses are external devices designed to support, stabilize, correct, or improve the function of a body part (usually the arm, leg, or spine).

**Purposes and Benefits of Orthoses in CP**

Orthoses are an important part of a **multidisciplinary treatment plan** for individuals with CP and serve multiple purposes:

- **Improve posture and alignment:** Help correct poor posture and joint alignment problems caused by muscle tone disorders (spasticity, hypotonia, etc.) or involuntary movements. This is especially important for ankle, knee, and hip joints.
- **Prevent or reduce muscle contractures:** In CP, muscles may shorten over time, limiting joint range of motion. Orthoses keep muscles in the correct position to prevent this shortening or slow its progression.
- **Increase stability and balance:** Provide stability for individuals with weak muscles or poor coordination, improving their ability to stand and walk, and reducing fall risk.
- **Improve gait pattern:** Especially ankle–foot orthoses (AFOs) can correct abnormal walking patterns like toe-walking, resulting in a smoother and more efficient gait.
- **Reduce pain:** Help relieve pain caused by poor posture or abnormal joint loading.
- **Increase functional independence:** Enable greater independence in daily activities such as eating, dressing, and walking.
- **Prevent or delay secondary deformities:** Early orthosis use can prevent permanent musculoskeletal deformities or delay the need for surgery.
- **Reduce energy expenditure:** Abnormal gait patterns require more energy; orthoses improve gait mechanics so the individual can stay active longer with less effort.

**Common Types of Orthoses in CP**

- **Ankle–Foot Orthosis (AFO):** Most common type; holds the ankle and foot in a set position for walking stability, corrects toe-walking, and prevents foot deformities. Variations include rigid and hinged types.
- **Knee–Ankle–Foot Orthosis (KAFO):** Supports the knee, ankle, and foot; used when there is knee instability or severe leg weakness.
- **Hand and Wrist Orthoses:** Maintain hand and wrist position, reduce spasticity, improve grip function, and prevent contractures.
- **Spinal Orthoses (Braces):** Used to manage spinal curvature (scoliosis) or support posture.

**Important Note:**

Orthosis selection and fitting must always be done by a **physical medicine and rehabilitation specialist** and an **orthotic technician**, in collaboration with the physiotherapy team.
As the child grows, orthoses should be **regularly checked and adjusted or replaced**.

**Question 7: What is botulinum toxin? Can it be used for my child with cerebral palsy? Are there any side effects?**

**What is Botulinum Toxin?**

Botulinum toxin is a powerful neurotoxin produced by the bacterium *Clostridium botulinum*.
When used in very small, controlled medical doses, it **temporarily blocks communication between nerves and muscles**, causing the targeted muscle to relax.
Because of this effect, it is used in the treatment of many conditions involving involuntary muscle contractions and spasms.
It is also widely known as “Botox” in cosmetic medicine for wrinkle treatment.

**Can It Be Used in My Child with CP?**

**Yes** — botulinum toxin is often and effectively used in children with CP.
Many children with CP have **spasticity** (muscle stiffness and excessive muscle tone), which limits movement, causes pain, makes daily activities difficult, and over time can lead to **permanent joint deformities** (contractures).

Botulinum toxin is injected into **specific muscle groups** to reduce spasticity.
It works by temporarily blocking the nerve signals that cause excessive contraction in that muscle, allowing it to relax.

**Benefits in Children with CP:**

- Reduces muscle stiffness and spasms → makes movement easier
- Increases range of motion → improves effectiveness of physiotherapy
- Corrects gait patterns (e.g., toe-walking)
- Makes daily activities like dressing, eating, and personal care easier
- Reduces pain caused by muscle tightness
- Delays or prevents the need for orthopedic surgery by slowing or stopping contracture progression

Botulinum toxin injections are generally used in **children over the age of 2** and last **3–6 months**.
Because the effect is temporary, treatment may need to be repeated at regular intervals.
It should always be planned and applied by a **pediatric neurologist** or **physical medicine and rehabilitation specialist**, tailored to the child’s individual condition.
It is usually combined with **physiotherapy and orthosis use** for best results.

**Are There Side Effects?**

**Common, Mild Side Effects:**

- Pain, redness, swelling, or bruising at the injection site (usually short-lived)
- Temporary muscle weakness in the treated muscle (rarely affects nearby muscles if dose is correct)
- Flu-like symptoms: fatigue, mild fever
- Headache in some children

**Rare but More Serious Side Effects:**

- **Toxin spread:** Very rarely, the toxin can spread beyond the targeted muscle, affecting nearby muscles (e.g., drooping eyelid, swallowing difficulty, breathing difficulty) — requires urgent medical attention
- Allergic reactions: itching, rash, shortness of breath (very rare)
- Antibody development: With repeated injections, the body may develop resistance, reducing effectiveness over time

**Important:** To minimize risks, botulinum toxin must be administered **by an experienced specialist**, in the correct dose, to the correct muscle.
Your doctor will weigh the risks and benefits based on your child’s overall health and needs.

**Question 8: Is surgery a possible treatment for cerebral palsy? When can surgery be performed?**

In Cerebral Palsy (CP), surgery **is possible**, but it is not a cure for the condition.
Instead, it is a treatment option aimed at **relieving symptoms, improving function, and enhancing quality of life**.
CP itself is caused by permanent brain damage, but surgery targets the **musculoskeletal problems** resulting from this damage.

**When is Surgical Treatment Considered in CP?**

Surgery is generally considered when **conservative (non-surgical) treatments** — such as physiotherapy, medication, or botulinum toxin injections — are insufficient, or when severe **spasticity** and deformities **significantly limit daily activities**.

In many cases, surgery is **avoided before the age of 7**, but this depends on the child’s individual needs and development.
Early intervention is important, but surgery is usually timed carefully.

**Goals of Surgery:**

- **Reduce muscle spasticity:** Relax overly tight muscles
- **Correct joint deformities:** Such as hip dislocation, knee or ankle deformities
- **Improve mobility and gait:** Help the child walk more steadily and independently
- **Relieve pain:** Reduce pain from muscle tightness or joint problems
- **Prevent or correct contractures:** Avoid long-term fixed muscle shortening and joint stiffness
- **Facilitate care:** Make personal hygiene and caregiving easier
- **Correct spinal deformities:** Such as scoliosis, especially in severe cases

**Types of Surgery in CP**

**1. Orthopedic Surgeries:**

- **Tendon Lengthening:** Lengthening shortened tendons (e.g., Achilles tendon, hamstrings) to improve joint range of motion
- **Tendon Transfers:** Moving tendons to improve function or balance
- **Osteotomy (Bone Correction):** Correcting bone deformities (e.g., hip or leg curvatures) and improving joint alignment
- **Joint Stabilization (Arthrodesis):** Fusing a joint in place to prevent excessive movement or deformity
- **Single Event Multilevel Surgery (SEMLS):** Addressing multiple muscle and bone issues in one surgical session

**2. Neurosurgical Procedures:**

- **Selective Dorsal Rhizotomy (SDR):** Cutting certain overactive sensory nerve fibers in the spinal cord to permanently reduce spasticity (requires intensive post-surgery physiotherapy)
- **Baclofen Pump Implantation:** For severe, widespread spasticity; delivers muscle relaxant (baclofen) directly into the spinal fluid via an implanted pump, allowing lower and more effective dosing

**How is the Decision Made?**

The decision for surgery should be made by a **multidisciplinary team**, including:

- Pediatric neurologist
- Orthopedic surgeon
- Physical medicine and rehabilitation specialist
- Physiotherapist

They will consider factors such as:

- Age
- Overall health
- Severity and distribution of spasticity
- Existing deformities
- Functional goals

**Post-Surgery:**

- Intensive **physiotherapy** is essential after surgery to make gains permanent and help the child reach maximum potential.

**Question 9: Can my child with cerebral palsy walk normally? When will they be able to walk?**

Whether a child with Cerebral Palsy (CP) can walk “normally” and when they might achieve walking depends on many factors, such as **the type of CP, severity, affected body areas, and the intensity of treatment**.
Every child with CP is different, and walking abilities can vary greatly.

**Can a Child with CP Walk Normally?**

“Normal walking” usually refers to the gait pattern of a typically developing child.
Some children with CP can come close to this, while others may have varying degrees of walking difficulty.

**Walking Ability Statistics:**

- **~60%** can walk independently — though gait may not be perfectly normal, they can move without assistance.
- **~10%** can walk with assistance — using walkers, canes, or support from another person.
- **~30%** require a wheelchair — due to more severe motor involvement.

**Gait Pattern Differences in CP:**

- Toe-walking
- Scissoring gait (legs crossing while walking)
- Bent-knee walking
- Circumduction gait (swinging the leg outward)

**Goal:** To make the gait pattern as functional as possible for independence.

**Importance of Rehabilitation**

Intensive, appropriate physiotherapy, orthotic support, botulinum toxin injections, and sometimes surgical interventions can **maximize walking potential**.
Early and consistent rehabilitation plays a key role in walking outcomes.

**When Can a Child with CP Walk?**

Walking ability is often assessed based on the **Gross Motor Function Classification System (GMFCS)**, which rates movement ability on 5 levels (Level I = most independent, Level V = most dependent).

**Mild Involvement (GMFCS Level I & II):**

- **Level I:** Walks independently around 18–24 months, similar to peers, with only minor limitations in running, jumping, or complex activities.
- **Level II:** Walks independently but may have difficulty on uneven surfaces, stairs, or in crowded areas. Usually walks by **age 3–4**, may need minimal support.

**Moderate Involvement (GMFCS Level III):**

- Can walk indoors or on flat surfaces with assistive devices (walker, crutches)
- May need a wheelchair for long distances
- Typically walks with assistance by **age 5–7**

**Severe Involvement (GMFCS Level IV & V):**

- **Level IV:** Usually able to sit with support, uses a wheelchair for mobility, may walk short distances with adult assistance or special walkers
- **Level V:** Needs full assistance for all mobility, uses a manual or powered wheelchair; independent walking not expected

**Key Points to Remember:**

- **Early Intervention:** Starting intensive physiotherapy in infancy can improve walking potential and possibly speed up the timeline.
- **Ongoing Monitoring:** A pediatric neurologist or rehabilitation specialist should regularly assess motor development and adjust the treatment plan.
- **Individual Differences:** Every child is unique — prognosis should come from the specialists who know the child’s progress best.

**Note:** Walking is an important goal for independence and quality of life in CP, but it is **not the only measure of success**. Communication, self-care, and social skills are equally important and should be part of a comprehensive rehabilitation plan.

**Question 10: What should I be careful about when dressing my baby with cerebral palsy or changing their diaper?**

When dressing or changing the diaper of a baby with Cerebral Palsy (CP), it’s important to consider the baby’s differences in motor development, muscle tone (tightness or looseness), and movement limitations.
Adopting special handling techniques will make the process more comfortable for both you and your baby.

**General Guidelines**

- **Stay Calm and Patient:**
  Babies with CP may have uncontrolled or restricted movements.
  Creating a calm environment and avoiding rushing reduces stress for both of you.
- **Talk to Your Baby:**
  Explain what you are doing — e.g., “I’m lifting your leg now” or “I’m putting on your shirt” — to make the process more predictable.
- **Observe Your Baby’s Reactions:**
  Learn which movements cause discomfort and which positions are easier. Every baby’s sensitivities are different.
- **Use Supportive Surfaces:**
  Work on a flat, safe surface such as a changing table or bed.
  Support your baby’s head, neck, and trunk, and use rolled towels or pillows for extra stability if needed.
- **Let the Baby Help (When Possible):**
  Depending on age and ability, allow your baby to assist with small movements, such as lifting an arm slightly. This encourages independence and participation.

**When Dressing**

- **Choose Front-Opening, Wide, and Stretchy Clothes:**
  - **Snap-button bodysuits and rompers:** Especially those that open fully at the front and between the legs make dressing and diaper changes easier.
  - **Wide necks and stretchy fabrics:** Avoid tight neck openings that are hard to pull over the head. Stretchy fabrics are more comfortable for babies with muscle stiffness.
  - **One size larger:** Slightly bigger clothes give more room for movement and make dressing easier.
- **Prefer Snaps Over Zippers and Buttons:**
  Zippers can pinch skin, and buttons can snag. Snaps are safer and quicker.
- **Gently Pass Arms and Legs Through Clothing:**
  - Avoid forcing joints.
  - If there is spasticity, slowly and gently straighten the arm or leg.
  - Slide your hand into the sleeve or pant leg first, then guide the baby’s limb through — this is easier than pushing the limb directly.
- **Layer Clothing:**
  Layering makes it easier to adjust body temperature by adding or removing clothes as needed.
- **Shoes and Socks:**
  Choose easy-to-wear shoes (Velcro or wide openings). Avoid very tight or lace-up shoes. Socks should be stretchy and comfortable.

**When Changing a Diaper**

- **Positioning:**
  - Lay the baby on their back.
  - Gently bend the legs upward or toward the tummy for cleaning — keep within the baby’s natural range of motion to avoid strain.
  - If needed, support the legs with one hand while cleaning with the other.
- **Be Quick and Prepared:**
  Keep all supplies (diaper, wipes, cream) within reach so you can complete the change quickly, reducing discomfort.
- **Skin Care:**
  Babies with CP may be at higher risk of diaper rash due to limited movement. Check the diaper area frequently, apply cream after cleaning, and make sure the skin is completely dry.
- **Adapt to Movement Limitations:**
  - If there is severe leg stiffness, separating the legs may be difficult — try cleaning with the baby in a side-lying position.
  - If trunk control is weak, support the body with one hand while changing with the other.
- **Safety:**
  Never leave the baby unattended on a changing table — sudden movements could cause a fall.

**Note:**
These are general guidelines. The best techniques depend on your baby’s individual needs.
Consult your baby’s **physiotherapist or pediatrician** for tailored advice and demonstrations.
